# Short-Term Feed Deprivation Alters Immune Status of Surface Mucosa in Channel Catfish (*Ictalurus punctatus*)

**DOI:** 10.1371/journal.pone.0074581

**Published:** 2013-09-04

**Authors:** Lisa Liu, Chao Li, Baofeng Su, Benjamin H. Beck, Eric Peatman

**Affiliations:** 1 Department of Fisheries and Allied Aquacultures, Auburn University, Auburn, Alabama, United States of America; 2 United States Department of Agriculture, Agricultural Research Service, Stuttgart National Aquaculture Research Center, Stuttgart, Arkansas, United States of America; 3 Department of Chemistry and Chemical Biology, College of Arts and Sciences, Cornell University, Ithaca, New York, United States of America; National Science and Technology Development Agency, Thailand

## Abstract

Short-term feed deprivation (or fasting) is a common occurrence in aquacultured fish species whether due to season, production strategies, or disease. In channel catfish (*Ictalurus punctatus*) fasting impacts susceptibility to several bacterial pathogens including *Flavobacterium columnare*, the causative agent of columnaris disease. As columnaris gains entry through the gills and skin of fish, we examined here changes in transcriptional regulation induced in these surface mucosal tissues due to short-term (7 day) fasting. RNA-seq expression analysis revealed a total of 1,545 genes perturbed by fasting. Fasting significantly altered expression of critical innate immune factors in a manner consistent with lower immune fitness as well as dysregulating key genes involved in energy metabolism and cell cycling/proliferation. Downregulation of innate immune actors such as iNOS2b, Lysozyme C, and peptidoglycan recognition protein 6 is predicted to impact the delicate recognition/tolerance balance for commensal and pathogenic bacteria on the skin and gill. The highlighted expression profiles reveal potential mechanistic similarities between gut and surface mucosa and underscore the complex interrelationships between nutrition, mucosal integrity, and immunity in teleost fish.

## Introduction

Alterations in feeding regimen are common practice in the modern aquaculture industry, predicated in part on research indicating that fasting is well tolerated by most fish species [1], [2]. Food deprivation strategies can be employed as part of a seasonal feeding pattern [3], in response to overproduction, or as a response to disease outbreak [4], [5]. When diseases prompt changes in feeding, producers must weigh potential savings on feed and the benefits of limiting fish-fish contact during feeding against reduced growth and heightened stress stemming from nutrient restriction. Traditionally missing in the equation has been any understanding on how short-term fasting may impact the immune status of farmed fish.

Most feed deprivation investigations to-date have focused on changes in intestinal cellular morphology and enzyme activity or on transcriptional changes in critical regulators of protein synthesis or glucose metabolism in tissues such as liver, muscle, and intestine [6]–[8]. Recent studies have documented changes in gene mediators of innate immunity following 4 week starvation studies in rainbow trout intestinal epithelia [9] and Atlantic salmon liver [10]. Comparatively little is known regarding the immune consequences of more common short-term fasting events. However, a small body of previous research indicates that short-term withdrawal of feed, while not visibly impacting growth parameters, can have striking effects, particularly at mucosal surfaces. Vieira et al. [11] reported broad impacts of a one week feed restriction regimen on skin healing and scale regeneration in sea bream utilizing an oligo microarray. Krogdahl and Bakke-McKellep [7] observed rapid 20–50% decreases in intestinal tissue mass and enzyme activities after two days of fasting in Atlantic salmon. Perhaps of greatest relevance here is the recent report that a seven day feed deprivation caused significant changes in microbial density and community composition in the cutaneous mucus of Atlantic salmon [12].

The impact of feed deprivation on mortality rates of channel catfish (*Ictalurus punctatus*) exposed to the bacterial pathogens *Edwardsiella ictaluri* and *Flavobacterium columnare* has previously been established [4], [5]. *F. columnare*, a widespread opportunistic pathogen of freshwater fish, and a leading cause of mortality in the U.S. catfish industry [13], causes higher losses in fish that have been withheld from feed for as little as seven days [4]. Attachment and entry of *F. columnare* into channel catfish is via surface mucosa, skin and gill. Recently, we identified a rhamnose-binding lectin (RBL) whose expression is strongly induced in channel catfish gill by *F. columnare* infection [14]. A more detailed subsequent study of RBL activity revealed that expression levels in gill were correlated with columnaris susceptibility, and that saturation of the receptor with its putative ligands resulted in significantly decreased columnaris mortality. Furthermore, RBL expression increased greater than 100-fold in the gill tissue of channel catfish fingerlings fasted for seven days [15]. Given these results and the impact of feeding status on columnaris susceptibility, we wished to examine further the broader molecular effects of short-term fasting of channel catfish on surface mucosal health. Towards that end, here we utilized RNA-seq-based transcriptome profiling of skin and gill homogenates from fed and 7 d fasted channel catfish fingerlings to better understand immune-nutritional regulation in teleost fish.

## Results

### Sequencing of short expressed reads from channel catfish gill and skin

A total of 209 million 100 bp high quality reads were generated for the fasted and fed samples. Greater than 26 million reads were generated for each of the six libraries. Raw read data are archived at the NCBI Sequence Read Archive (SRA) under Accession **SRP017689**.

### *De novo* assembly of channel catfish gill and skin transcriptome

Given the importance of assembly of long, accurate contigs to capture channel catfish genes and to correctly identify differential expression, we compared two prominent options for *de novo* transcriptome assembly: Trans-ABySS and Trinity. We had previously developed an in-house bioinformatics pipeline around Trans-ABySS [14], [16] and demonstrated its superior performance in comparison to use of CLC Genome Workbench or Velvet assemblers. However, we sought to determine whether use of Trinity [17] would improve assemblies further.

#### Trans-ABySS

Use of Trans-ABySS to merge ABySS multi-k-assembled contigs, resulted in approximately 301,500 contigs with average length of 1,036.1 bp and N50 size of 1,716 bp, with 24,345 contigs longer than 1,000 bp. After removing redundancy using CD-Hit and CAP-3, about 57.53% contigs were kept, resulting in a final assembly of 173,459 unique contigs with average length 887.3 bp (Table 1).

**Table 1:** Summary of *de novo* assembly results of Illumina RNA-seq data from channel catfish gill and skin using Trans-ABySS and Trinity assemblers.

	Trans-ABySS	Trinity
Contigs	301,500	281,595
Large contigs (≥1000 bp)	24,345	30,202
N50 (bp)	1,716	2,270
Average contig length	1,036.1	1046.9
Contigs (After CD-HIT + CAP3)	173,459	272,229
Percentage contigs kept after redundancy removal	57.6%	96.7%
Average length (bp) (After CD-HIT + CAP3)	887.3	996.5
Reads mapped in pairs (%)	66.3%	79.9%
Reads mapped to final reference(%)	80.8%	85.7%

#### Trinity

Trinity generated approximately 281,595 contigs in its initial contig assembly with average length of 1,046.9 bp and N50 size of 2,270 bp, with 30,202 contigs longer than 1,000 bp. After removing redundancy, about 96.67% contigs were kept, resulting in a final 272,229 contigs with average length 996.5 (Table 1).

#### Gene identification and annotation

BLAST-based gene identification was performed to annotate the channel catfish gill/skin transcriptome and inform downstream differential expression analysis. After gene annotation, 60,892 contigs from the Trans-ABySS assembly had significant BLAST hits against 16,610 unigene (unique gene) matches from zebrafish, the closest available reference genome to channel catfish (Table 2). Using a more stringent criteria of a BLAST score ≥100 and E-value ≤ 1e-20 (quality matches) identified 14,679 zebrafish unigene matches. The same BLAST criteria were used to annotate the Trans-ABySS assembly based on matches against the UniProt and NCBI nr (non-redundant) databases.

**Table 2:** Summary of gene identification and annotation of assembled catfish contigs based on BLAST homology searches against various protein databases (Zebrafish, UniProt, nr).

	Trans-ABySS			Trinity		
	Zebrafish	UniProt	nr	Zebrafish	UniProt	nr
Contigs with putative gene matches	60,892	50,766	64,788	82,365	68,797	83,972
Annotated contigs ≥500 bp	41,313	37,468	44,019	71,782	61,658	73,849
Annotated contigs ≥1000 bp	29,023	27,479	30,379	59,986	53,210	61,185
Unigene matches	16,610	19,341	25,416	17,892	19,970	26,549
Hypothetical gene matches	986	0	3,788	1,058	0	3,693
Quality Unigene matches	14,679	15,933	15,863	15,821	15,427	20,589

Putative gene matches were at E-value ≤ 1e-5. Hypothetical gene matches denote those BLAST hits with uninformative annotation. Quality unigene hits denote more stringent parameters, including score ≥100, E-value ≤ 1e-20.

In contrast, 82,365 Trinity contigs had a significant BLAST hit against 17,892 unique zebrafish genes (Table 2). 15,821 unigenes were identified based on hits to the zebrafish database with the more stringent criteria of a BLAST score ≥100 and E-value ≤ 1e-20. As with the Trans-ABySS assembly, the same BLAST criteria were used in comparison of the Trinity reference contigs with the UniProt and nr databases. The largest number of matches was to the nr database with 83,972 contigs with putative gene matches to nr and 20,589 quality unigene matches (Table 2).

#### Best assembly selection

In a comparison of the assemblies generated by Trans-ABySS and Trinity (Table 1), it was clear that although Trans-ABySS consistently generated a larger initial number of contigs, redundancy was much higher than that observed with Trinity. CD-HIT/CAP3 removed over 40% of Trans-ABySS contigs due to this redundancy, while almost all Trinity contigs were carried forward after this process. The final Trinity assembly contained almost 100,000 more contigs than Trans-ABySS due to Trinity's superior ability to distinguish splicing isoforms and gene paralogs. Trinity contigs also had larger N50 and average length, 2,270 bp and 996.5 bp, respectively, than Trans-ABySS. These metrics reflect Trinity's superior ability to map paired end reads into the same contig. Trinity mapped 79.9% of reads in pairs versus 66.3% in Trans-ABySS (Table 1). The superior de novo assembly produced by Trinity was also reflected in the number of quality unigene matches against zebrafish (15,821 vs. 14,679) and nr (20,589 vs. 15,863) databases. Given these results, we utilized the Trinity assembly for subsequent analysis of differential expression.

### Identification and analysis of differentially expressed genes

A total of 1,545 genes (unique annotated contigs with significant BLAST identities) were differentially expressed greater than 1.5-fold, with 412 up-regulated genes and 1,133 down-regulated genes (Table 3; Table S2.). Short read coverage within differentially expressed contigs is critical for accurate quantification of expression. We obtained good coverage of differentially expressed contigs, with an average of 784.6 reads/contig.

**Table 3:** Statistics of differently expressed genes between fasted and fed channel catfish with fed catfish serving as the control.

Genes	Fasted Channel Catfish
Up-regulated	412
Down-regulated	1,133
Total	1,545
Reads per contig	784.6

Values indicate contigs/genes passing cutoff values of fold change ≥1.5 (p<0.05). Average contig size refers to reads/contig.

### Enrichment and pathway analysis

A total of 3,482 GO terms including 970 (27.86%) cellular component terms, 1,012 (29.06%) molecular functions terms and 1,491 (42.82%) biological process terms were assigned to 1,545 unique gene matches. The percentages of annotated channel catfish sequences assigned to GO terms are shown in Fig. S1. The differently expressed unique genes were then used as inputs to perform enrichment analysis using Ontologizer. A total of 125 terms with p-value (FDR-corrected) <0.05 were considered significantly overrepresented. Ten higher level GO terms were retained as informative for further pathway analysis (Table S3). The GO terms included response to stress, regulation of cell death, and cell cycle regulation.

Based on enrichment analysis and manual annotation and literature searches, representative key genes were arranged into three broad categories, including immune response, energy metabolism, and cell cycling (Table 4). Imputed putative functional roles of these genes are covered in the Discussion.

**Table 4:** Key differentially expressed genes in the gill and skin between fasted and fed channel catfish in different functional classifications.

Gene Name	Contig_ID	Fold Change
**Immune (Reads Number >0)**		
Autoimmune regulator-like	comp99702_c0_seq1	−2.08
Beta-2-glycoprotein 1	comp100784_c1_seq4	9.82
cAMP-responsive element modulator	comp90058_c0_seq2	−6.47
CC chemokine SCYA113	comp82613_c0_seq1	−4.30
C-C motif chemokine 19-like precursor	comp81887_c0_seq1	−2.63
CD83	comp88858_c0_seq1	1.82
Chemokine CCL-C5a precursor	comp33700_c0_seq1	−3.30
Clusterin precursor	comp33405_c0_seq1	3.57
Complement C1q tumor necrosis factor-related protein 6	comp99810_c0_seq1	−3.04
Complement C4-B	comp99246_c0_seq3	4.40
C-X-C motif chemokine 10 precursor	comp90838_c0_seq1	−3.61
C-X-C motif chemokine 11-like	comp100129_c0_seq1	−3.46
Eosinophil peroxidase precursor	comp98419_c0_seq2	−2.14
Galectin-4-like isoform 1	comp93775_c0_seq1	−1.80
IgM chain C region, secreted form - channel catfish	comp94145_c1_seq2	−1.69
IgGFc-binding protein-like	comp100810_c0_seq1	−2.62
Immunoresponsive gene 1, like	comp55693_c0_seq1	−53.66
Interferon-induced protein 44	Contig6350	3.46
Interferon-induced very large GTPase 1-like	comp102878_c0_seq1	1.73
Interleukin 17a/f1 precursor	comp102117_c4_seq2	−3.27
Interleukin-22 receptor subunit alpha-2 precursor	comp77551_c0_seq1	−2.13
Intestinal-type alkaline phosphatase precursor	comp60600_c0_seq1	90.69
Lymphokine-activated killer T-cell-originated protein kinase	comp89030_c0_seq1	−2.99
Lysozyme G	comp91870_c0_seq1	−2.78
Lysozyme G-like 1	comp91870_c0_seq2	−2.07
Lysozyme C	comp75157_c0_seq1	−6.35
Matrix metalloproteinase-19-like	comp100787_c0_seq1	−1.74
MHC class II antigen	Contig2104	2.13
MHC class II beta chain	comp101401_c0_seq1	1.91
Microfibril-associated glycoprotein 4-like	comp87348_c0_seq1	−5.74
Myxovirus (influenza virus) resistance G	Contig5990	−2.95
NACHT, LRR and PYD domains-containing protein 14-like	comp95945_c1_seq1	1.64
NADPH oxidase organizer 1a	comp100364_c0_seq1	−1.81
Nitric oxide synthase 2b, inducible (iNOS2b)	comp93125_c0_seq1	−17.22
Olfactomedin-like	comp100788_c0_seq4	−2.08
Peptidoglycan recognition protein 6 (PGRP6)	comp88793_c0_seq1	−9.13
Polymeric immunoglobulin receptor (pIgR)	comp93688_c0_seq2	−1.70
Protein canopy homolog 2 precursor	comp93948_c0_seq1	−3.35
SAM domain and HD domain-containing protein 1-like	comp98561_c0_seq8	6.64
Serum amyloid P-component precursor	comp91375_c0_seq2	1.69
Toxin-1 precursor	comp112912_c0_seq1	−6.89
Vitelline membrane outer layer protein 1 homolog	comp101161_c0_seq1	−3.55
Complement C1q 3-like	comp85071_c0_seq1	−3.41
**Immune (Reads Number contain 0)**		
Chitinase, acidic.1 precursor	comp84271_c0_seq1	0/84
Chitinase, acidic.3 precursor	comp72677_c0_seq1	0/51
**Energy Metabolism**		
6-phosphofructo-2-kinase/fructose-2,6-biphosphatase 4	comp97436_c0_seq2	2.77
Adipocyte enhancer-binding protein 1	comp97921_c0_seq1	3.26
Angiopoietin-related protein 4 precursor	comp95719_c0_seq1	3.16
Apolipoprotein Bb precursor	comp99989_c0_seq2	54.62
Apolipoprotein E precursor	comp53599_c0_seq1	3.80
Apolipoprotein L, 1	comp96519_c0_seq1	2.81
Carboxypeptidase A6-like	comp510161_c0_seq1	10.95
Carnitine O-acetyltransferase-like	comp97193_c0_seq1	2.35
Carnitine O-palmitoyltransferase 1, liver isoform	comp98534_c0_seq8	6.12
Corticosteroid 11-beta-dehydrogenase isozyme 2	comp84850_c0_seq1	5.72
Cytoplasmic phosphatidylinositol transfer protein 1	comp98091_c0_seq1	4.28
Diacylglycerol O-acyltransferase 2	Contig576	−5.78
Endothelial lipase precursor	comp118134_c0_seq1	−3.40
Fat storage-inducing transmembrane protein 2	comp85823_c0_seq1	−2.31
Fatty acid binding protein 1-B.1	comp143596_c0_seq1	11.26
Fatty acid-binding protein, brain	comp72920_c0_seq1	−3.57
Fatty acid-binding protein, intestinal	comp91705_c1_seq1	29.35
F-box only protein 32	comp83869_c0_seq1	9.07
Fructose-1,6-bisphosphatase 1b	comp99650_c0_seq4	3.96
Glutamine synthetase	comp90753_c0_seq1	−1.76
Glyceraldehyde 3-phosphate dehydrogenase 2	comp86158_c0_seq4	−2.03
Glycogen synthase 1	comp91600_c0_seq1	2.51
Growth hormone receptor b precursor	comp84382_c0_seq2	2.88
Hexokinase-2	comp93533_c0_seq1	−2.98
Krueppel-like factor 15	comp98940_c1_seq5	2.82
Lipoprotein lipase precursor	comp97781_c0_seq1	−2.15
Malate dehydrogenase	comp86729_c0_seq2	2.57
Mannose-1-phosphate guanyltransferase alpha-B	comp95181_c0_seq1	−5.29
Muscle RING finger 1	comp59139_c0_seq1	2.72
Probable fructose-2,6-bisphosphatase TIGAR A	comp91599_c0_seq3	−5.35
Relaxin-3 receptor 1-like	comp82094_c0_seq1	3.70
Solute carrier family 15 member 1	comp98443_c5_seq1	5.36
Solute carrier family 2, facilitated glucose transporter	comp91908_c0_seq2	−3.54
Squalene synthase	comp97820_c0_seq5	2.69
Star-related lipid transfer protein 4	comp102843_c2_seq1	−9.75
Stearoyl-coa desaturase 5	comp96892_c0_seq3	−8.22
Pyruvate dehydrogenase kinase, isozyme 4	comp101992_c0_seq2	5.99
**Cell Cycling/Proliferation**		
Anterior gradient protein 2 homolog precursor	comp74849_c0_seq1	−3.33
Antigen KI-67-like	comp93863_c1_seq1	−3.10
Borealin	comp98879_c0_seq1	−3.18
Cell division control protein 2 homolog	comp111617_c0_seq1	−3.88
Cell division cycle-associated protein 2	comp83275_c0_seq1	−3.08
Cell division cycle-associated protein 3	comp93142_c0_seq1	−3.18
Cell division cycle-associated protein 7	comp100916_c4_seq2	−2.89
Cyclin-A2	comp96163_c2_seq2	−3.71
Cyclin-dependent kinase inhibitor 1C-like	comp92450_c0_seq1	3.18
Cyclin-dependent kinase inhibitor 3	comp85776_c0_seq1	−2.89
DNA replication licensing factor MCM3	comp92554_c0_seq1	−3.36
DNA replication licensing factor MCM4	Contig155	−2.92
DNA replication licensing factor MCM5	comp85130_c0_seq1	−3.14
DNA replication licensing factor MCM6	comp88864_c0_seq1	−3.42
Eukaryotic translation initiation factor 2-alpha	Contig6286	−1.94
G1/S-specific cyclin-E2	comp82150_c0_seq2	−3.78
G2/mitotic-specific cyclin-B1	comp80016_c0_seq1	−3.63
G2/mitotic-specific cyclin-B3	comp102297_c0_seq2	−2.39
Mitotic checkpoint serine/threonine-protein kinase BUB1	comp94936_c0_seq1	−3.49
PCNA-associated factor-like	comp115121_c0_seq1	−2.52
Protein regulator of cytokinesis 1	comp92273_c0_seq4	−7.87
Structural maintenance of chromosomes 2	comp101484_c0_seq1	−3.01

Positive/negative values indicate upregulation and downregulation, respectively, in fasted fish relative to the fed control. When reads number equaled to 0 in fed or fasted group, the fold change is presented by average normalized read number in fed/average normalized read number in fasted. All fold changes were significant at p-value <0.05.

### Validation of RNA-seq profiles by QPCR

**Figure 1:**
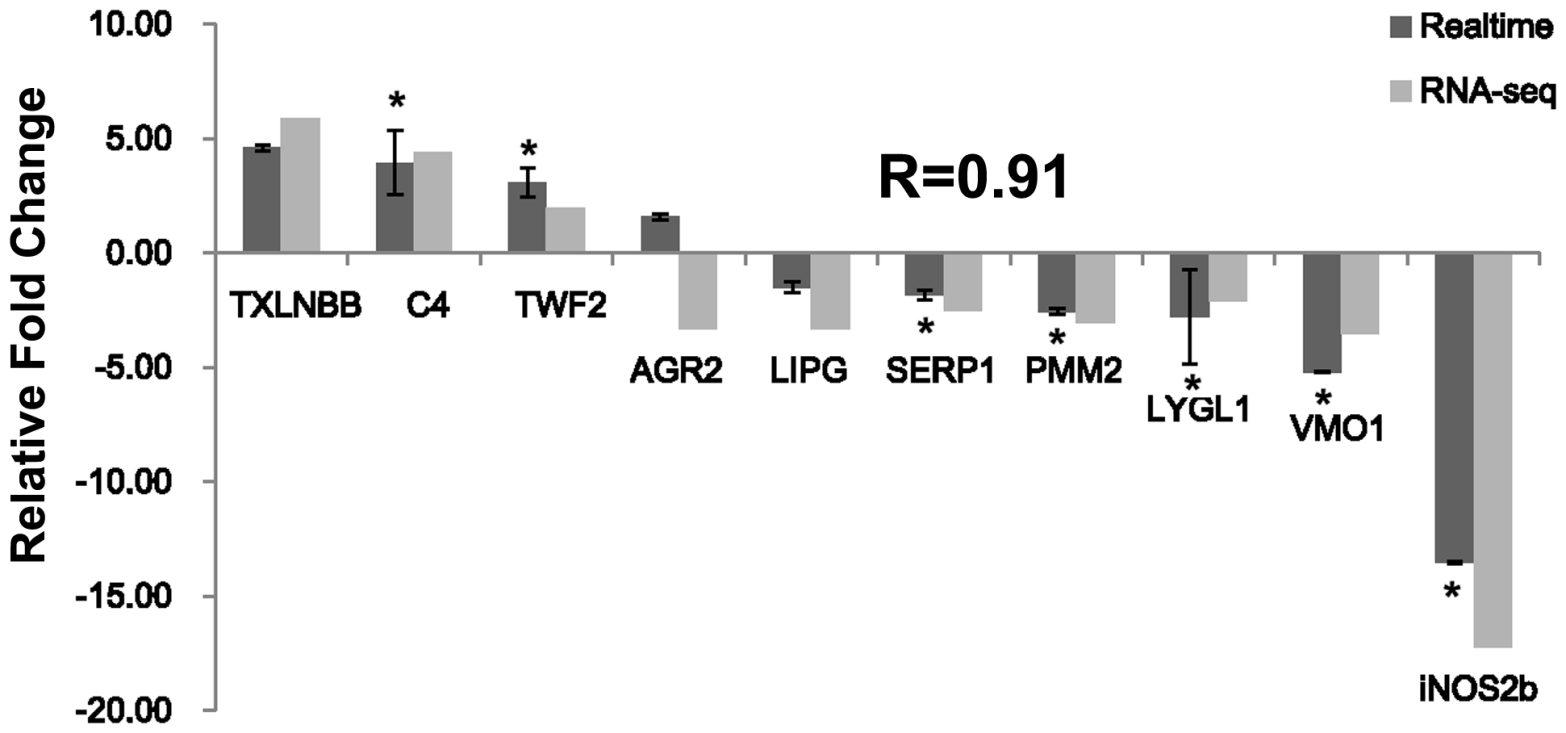
Comparison of relative fold changes between RNA-seq and QPCR results in channel catfish. Gene abbreviations are: Lysozyme g-like 1, LYGL1; Taxilin beta b, TXLNBB; Twinfilin-2, TWF2; Endothelial lipase precursor, LIPG; Stress-associated endoplasmic reticulum protein 2, SERP1; Anterior gradient protein 2 homolog precursor, AGR2; Phosphomannomutase 2, PMM2; Complement c4, C4; Nitric oxide synthase 2 b, inducible, iNOS2b; Vitelline membrane outer layer protein 1 homolog, VMO1.

We selected 10 genes for QPCR confirmation, choosing from those with differing expression patterns and from genes of interest based on functional enrichment and pathway results. Samples from fasted and fed channel catfish (with three replicate sample pools per timepoint) were used for QPCR. Melting-curve analysis revealed a single product for all tested genes. Fold changes from QPCR were compared with the RNA-seq expression analysis results. As shown in Fig. 1, QPCR results were significantly correlated with the RNA-seq results (average correlation coefficient 0.91, p-value <0.001; Figure 1). With the exception of anterior gradient protein 2 (AGR2), all examined genes had the same direction of differential expression by both methods indicating the reliability and accuracy of the Trinity reference assembly and RNA-seq-based transcriptome expression analysis.

## Discussion

While it is well understood that nutritional perturbations can modulate resistance to infectious diseases in agricultural animals [18], the molecular mechanisms by which dietary changes alter fish host immunity are largely unknown. The impact of these changes would be expected to differ depending on duration of feed withdrawal (fasting vs. starvation), species-specific evolutionary adaptation to natural levels of feed availability, season, and pathogen dynamics (prevalence, routes of infection, etc). Short (one week) periods of feed deprivation in channel catfish can decrease mortality rates to a Gram-negative bacterial pathogen, *E. ictaluri* [5], but, conversely, appear to lead to higher mortality to *F. columnare* [4]. Given that *F. columnare* gains entry through surface mucosa, we examined here transcriptomic changes in channel catfish gill and skin following a 7 d feed deprivation challenge. RNA-seq analysis revealed a total of 1,545 genes with expression perturbed by fasting. Fasting significantly altered expression of critical innate immune factors in a manner consistent with lower immune fitness as well as dysregulating key genes involved in energy metabolism and cell cycling/proliferation.

Studies of feed deprivation have traditionally focused on direct impacts on the digestive tract [19] or on systemic alterations in energy homeostasis [20]. Our understanding of the importance of mucosal surfaces as the front line in host immunity and the primary target for pathogen invasion continues to grow [21], [22]. However, the sensitivity of mucosal defensive barriers to nutritional changes is only now being explored in fish [12], [23]. Advances in next-generation sequencing now allow rapid, comprehensive analysis of the molecular underpinnings of these phenomena through RNA-seq approaches [24]. In order to gain a perspective on the molecular actors in the surface mucosa responding to fasting we pooled equal amounts of skin and gill from each fish contributing to the replicated tissue pools utilized for RNA-seq analysis. Clearly, by pooling heterogeneous cell populations stemming from the two tissues the potential exists for masking or confusing gene expression patterns from particular cellular subsets. However, we accepted this compromise in this initial study to more broadly capture novel genes and patterns in the surface mucosa. Future research will seek to ascertain which tissues and constituent cell types are contributing to key transcript profiles using laser capture micro-dissection and antibody-based cell sorting.

A total of 1,545 differentially expressed contigs could be annotated based on BLAST analysis (Table S2). We attempted to classify key differentially expressed genes into broad functional categories based on GO annotation and manual imputation of putative function via literature search of studies in vertebrate model organisms (Table 4). Below we highlight several important pathways likely mediating the channel catfish mucosal response to short-term (7 d) fasting.

### Immune Function

Recent work by our group examined how the gill transcriptome differed between *F. columnare* resistant and susceptible channel catfish both basally and at early timepoints following infection [25]. That dataset, combined with recent analysis of pathogen-mediated changes in the catfish skin and intestinal transcriptomes [16], [26], [27], provided a foundational reference of genes likely modulating immunity in mucosal tissues useful for comparison here. Initial analysis revealed enrichment of genes with immune functions among the mostly highly differentially expressed genes following fasting. Of the top 15 upregulated genes following fasting, 11 had immune functions according to a combination of GO annotation and manual literature searches. Similarly, 6 of the 15 most highly downregulated genes also had known immune functions. Among immune genes perturbed by fasting were acidic chitinase precursor genes which were not sequenced in fed fish (0 reads), but were present with between ∼30–125 reads in the fasted replicates. Previously, we examined zero read data and concluded that although fold changes are difficult to predict, these data reflect true differential expression [25]. Chitinases have received little attention in fish to-date and have not been reported in the skin or gill of any species. However, in mammals, chitinases have the ability to cleave inhaled or ingested chitins from fungi, parasitic worms, crustaceans, and insects and are known to participate in the pathogenesis of allergic inflammation, particularly in the lung [28]. The acidic mammalian chitinase (AMCase) in particular has been found to be causative in lung inflammation and induced by IL-13 [29]. Also induced by fasting was an intestinal-type alkaline phosphatase (IAP; 90.69-fold). Known as a critical brush-border protein in mammals, its functions and expression patterns in fish have not been previously characterized. In mammals, IAP has the ability to detoxify lipopolysaccharide (LPS) and prevents infection across the gut mucosal barrier. Additionally, as here, IAP is reported to be sensitive to nutritional changes [30], [31]. However, IAP was silenced in the gut after 2 days of fasting in mice [30], contrasting with the induced expression seen here in skin and gill samples at 7 d. Clearly, additional study is required to delineate novel roles of IAP in modulating mucosal immune events in teleost fish.

Also induced following fasting were several genes whose expression had been perturbed in fish mucosa in previous studies in channel catfish. These included beta-2-glycoprotein 1, a novel mediator of innate immunity recently discovered to interact with LPS and activate macrophages [32]. We previously observed sharp upregulation of beta-2-glycoprotein 1 at 2 h after *Aeromonas hydrophila* infection of channel catfish skin [27]. Interestingly, beta-2-glycoprotein 1 was also reported to be upregulated following 7 d fasting in sea bream skin. Other examples such as SAM domain and HD domain-containing 1 (SAMHD1) and serum amyloid-P precursor (SAP) were induced here by fasting and upregulated in the gill following columnaris challenge [14], [25].

A suite of immune genes were also strongly downregulated following fasting (Table 4). Immunoresponsive gene 1 (IRG1), for example, was downregulated over 53-fold after the 7 d fasting period. IRG1 is a LPS-inducible gene linked to susceptibility to Marek's Disease in chickens [33]. Additionally, we found basally lower levels of IRG1 in *F. columnare* susceptible channel catfish gill when compared with resistant channel catfish [25]. Also among downregulated immune genes following fasting was inducible nitric oxide synthase 2 b (iNOS2b), previously established as the most likely orthologue of mammalian iNOS [34]. iNOS generates nitric oxide (NO) from L-arginine. NO is a potent cytotoxic agent in immune defenses which can have beneficial antimicrobial activity at mucosal surfaces including respiratory epithelium [35]. Previously, we reported high constitutive expression of iNOS2b in healthy channel catfish gill and higher iNOS2b levels in *F. columnare* resistant catfish than susceptible channel catfish [25]. Here, following 7 d fasting, iNOS2b transcript levels plunged greater than 17-fold. Based on our previous studies, we would predict that these fasted levels of iNOS2b may open up channel catfish to heightened levels of pathogen colonization.

Another noteworthy alteration in immune status induced by food deprivation was the downregulation of peptidoglycan recognition protein 6 (PGRP6; -9.13-fold). Peptidoglycan recognition proteins are poorly-studied pathogen recognition molecules which can bind and kill commensal and pathogenic bacteria, often upstream of the better characterized Toll pathway [36]. Host species from insects to mammals rely on a diverse array of PGRP molecules, some mucus-secreted, to shape and coordinate responses to a wide range of microorganisms. They appear particularly adapted to local responses at mucosal epithelial interfaces rather than more systemic immune responses [37]. For example, in Drosophila PGRP proteins mediate tolerance of gut epithelia toward endogenous microbiota [38]. PGRP6 is one of four PGRP genes in zebrafish. Suppression of PGRP6 by RNAi in zebrafish downregulated the Toll pathway and resulted in markedly increased susceptibility to *F. columnare* [39]. Taken together, these studies point to the potential importance of PGRP6 as a nutrition-sensitive mediator of mucosal health. Its presence at the mucosal surface in fish may be important for the establishment of tolerance to commensal microbial populations through regulation of release of appropriate levels of antibacterial factors such as mucins and iNOS [37]. Future work should further characterize PGRP6 activity in catfish in the context of nutrition and disease.

In our previous study of patterns of gene expression linked to *F. columnare* resistance, levels of lysozyme C in channel catfish gill were markedly higher both pre-challenge and following infection in resistant fish [25]. Here, fasting for 7 d decreased lysozyme C levels greater than 6-fold. In mammals, lysozymes are critical mucosal enzymes secreted from the epithelium as well as a major component of granules of professional phagocytes. They help to kill bacterial pathogens through enzymatic and antimicrobial activity [40]. Previous research in zebrafish, in which a transgenic zebrafish strain expressing a chicken lysozyme gene under the control of a keratin promoter resulted in a 65% survival rate against *F. columnare* compared to 0% survival in wild-type fish, also points to the importance of lysozyme in fish mucosal immunity [41]. Additional genes exhibiting co-regulation in both the context of feed deprivation and *F. columnare* immunity included autoimmune-regulator like, CCL19, CD83, IgFc-binding protein-like, IL-17A, MFAP4, polymeric immunoglobulin receptor (pIgR), and toxin-1 precursor (Table 4; [25]). The fasting-induced modulation of genes previously indicated to be critical in resistance to *F. columnare* may explain in part the heightened sensitivity to *F. columnare* observed in fasted fish [4]. Fasting appears to decrease levels of innate immune mediators including iNOS2b, Lysozyme C, and PGRP6 at the surface barriers where *F. columnare* gains entry.

### Energy Metabolism

While our primary focus here was to examine direct impacts of short-term feed deprivation on immune regulation in channel catfish surface mucosa, we also examined broader physiological impacts on cellular health and function. Changes in metabolic or stress parameters may be important indicators of reallocation of energy reserves and/or changes in barrier permeability which may, in turn, impact host pathogen susceptibility [18], [42]. Among the most highly induced genes following fasting were several apolipoproteins. Apoliproteins are proteins which classically bind and transport lipids through the blood but are also recognized for roles in immunity and acute phase responses [43]. Greatest changes were seen in the apolipoprotein ApoBb (Table 4). ApoB serum levels in humans have been reported to rise following fasting [44]. ApoE and ApoL1 levels also rose in channel catfish skin/gill following fasting, contrasting with starvation-induced decreases of apolipoproteins in the liver of rainbow trout and salmon [10], [45].

Expression of several fatty-acid binding proteins (FABP) were perturbed by fasting (Table 4), indicating changes in lipid metabolism. Largest changes were observed in the intestinal FABP, FABP2, which was induced greater than 29-fold. FABPs are well studied as intracellular fatty acid transporters in vertebrates, with limited studies to-date in fish [46]. Better studied in fish are genes linked to proteolytic gene expression during muscle atrophy [47], [48]. The ubiquitin ligases F-box only protein 32 (Fbx32) and muscle RING finger 1 (MuRF1) were upregulated following fasting, 9.07-fold and 2.72-fold respectively.

The absorption of oligopeptides following protein digestion in vertebrates is facilitated by a member of the solute carrier 15 family, SLC15A1 or PepT1. In our results, the transporter was upregulated 5.36-fold in channel catfish following fasting. Given that most luminal products of protein digestion in fish are di- and tripeptides rather than individual free amino acids, PepT1 is thought to be the major transporter of ingested protein across the gut mucosa [49]. Considerable research has been directed at understanding PepT1 function in a range of vertebrates including fish [50]. Studies in several teleost species have revealed that intestinal expression levels of PepT1 respond to fasting and refeeding, with expression levels rising in some species after short-term fasting, followed by a decline in long-term fasting/starvation. Re-feeding stimulates upregulation of PepT1 above pre-fasted levels, indicating sensitive responsiveness to food availability and a potential role for the transporter in the phenomenon known as compensatory growth [51]–[54]. Aquaculture researchers are keenly interested in use of PepT1 as an indicator of protein uptake and as a direct predictor of animal growth [51], [55]. PepT1 may also serve as another bridge between nutritional and immune regulation, given its roles in binding bacterial peptides and stimulation of gut innate immune activation and inflammation [56]. However, all the aforementioned studies focus on PepT1 function in the intestine. While Terova et al. [54] reported second highest tissue expression of PepT1 in sea bass gills (behind some but not all intestinal segments), no previous reports of a PepT1 response to fasting in teleost surface mucosa exist. Given the importance of skin and/or gill epithelia for nutrient absorption in invertebrates and at least one ancient vertebrate, the hagfish [57], [58], one is tempted to speculate that catfish PepT1 fasting-induced expression in the skin/gill may reflect conserved mechanisms of nutrient sensing and/or uptake previously unknown in fish. Further research is needed to pinpoint cellular sources of PepT1 expression in surface mucosa and examine responses to longer-term fasting and re-feeding.

### Cell Cycling/Proliferation

A final notable category of genes perturbed in channel catfish skin/gill following short-term fasting were those involved in cell cycling and proliferation. Again here, while no previous study has examined how fasting may impact cell division in fish surface mucosa, our understanding of these processes in the mammalian gastrointestinal mucosa is fairly well refined [59]. There, fasting/starvation has been found to be one of the best models of a hypoproliferative response [60]. Subsequent studies have established a consistent pattern of feed deprivation reducing proliferation rates across multiple cell types, such that fasting and calorie restriction are now recommended as approaches to impede tumor growth [61]. We observed consistent downregulation of a large number of genes involved in cell cycling, proliferation, and differentiation (Table 4 and Table S2). These included Ki67 and PCNA-associated factors from channel catfish, cyclins, DNA replication licensing factors and checkpoint proteins (Table 4). The alteration of cell cycling indicated by these transcript changes may disrupt the integrity of catfish mucosal barriers with consequences for immune defenses at these surfaces [62].

## Conclusion

RNA-seq-based transcriptome profiling in channel catfish revealed that short-term feed deprivation altered immune status in the surface mucosa. Changes in innate immune actors such as iNOS2b, Lysozyme C, and PGRP6 may impact the delicate recognition/tolerance balance for commensal and pathogenic bacteria on the skin and gill. Furthermore, our analysis identified critical regulators of metabolism, cell cycling, and transport previously unstudied and/or unreported in these tissues whose expression was perturbed by fasting. The highlighted expression profiles reveal potential mechanistic similarities between gut and surface mucosa and underscore the complex interrelationships between nutrition, mucosal integrity, and immunity in teleost fish.

## Materials and Methods

### Experimental animals and tissue collection

Animal care and experimental protocols were approved by the Stuttgart National Aquaculture Research Center Institutional Animal Care and Use Committee and conformed to Agricultural Research Service Policies and Procedures 130.4 and 635.1. Juvenile channel catfish (42.2±5.6 g) were stocked into four 600 L tanks with 30 fish per tank with forced air aeration and flow-through well water at 24.8±0.02°C, pH 7.7, and dissolved oxygen of 7.4±0.3 mg/L.

Channel catfish were subjected to two treatments with two replicate tanks per treatment. Fish in treatment group 1 were fed to satiation three times daily with a standard catfish ration (35% protein, 2.5% fat). In treatment 2, fish were withheld feed for 7 d. All fish were sacrificed on day 7, euthanized with tricaine methanesulfonate (MS 222) at 300 mg/L before tissues were collected.

Equivalent portions of the gill and skin were isolated from matching locations on each fish and stored in RNALater (Ambion, Life Technologies, Grand Island, New York) at −80°C until RNA extraction. Three pools (five fish each) of tissue were generated from each condition. Equal amounts of tissue were collected from each fish within a pool. Samples were immediately placed in RNAlater and stored at −80°C until extraction. Samples were homogenized with a mortar and pestle.

### RNA extraction, library construction and sequencing

Extractions were performed according to the manufacturer's directions using an RNeasy Kit (Qiagen, Valencia, California). RNA concentration and integrity of each sample was measured on an Agilent 2100 Bioanalyzer. For each condition, equal amounts of RNA from skin and gill were pooled to generate three pooled replicates for RNA-seq library construction.

RNA-seq library preparation and sequencing was carried out by HudsonAlpha Genomic Services Lab (Huntsville, AL, USA) following the standard TruSeq protocols with 100 bp PE read chemistry on an Illumina HiSeq 2500 instrument [14].

### *De novo* assembly of sequencing read

The *de novo* assembly of short reads was performed on channel catfish using both ABySS and Trinity [17], [63], versions 1.3.2 and the 2012-10-05 editions, respectively. Before assembly, raw reads were trimmed by removing adaptor sequences and ambiguous nucleotides. Reads with quality scores less than 20 and length below 30 bp were all trimmed. The resulting high-quality sequences were used in the subsequent assembly.

In ABySS, briefly, the clean reads were first hashed according to a predefined k-mer length, the ‘k-mers’. After capturing overlaps of length k-1 between these k-mers, the short reads were assembled into contigs. The k-mer size was set from 50 to 96, assemblies from all k-mers were merged into one assembly by Trans-ABySS (version 1.4.4).

In Trinity, briefly, the raw reads were assembled into the unique sequences of transcripts in Inchworm via greedy k-mer extension (k-mer 25). After mapping of reads to Inchworm contigs, Chrysalis incorporated reads into deBruijn graphs and the Butterfly module processed the individual graphs to generate full-length transcripts.

In order to reduce redundancy, the assembly results from different assemblers were passed to CD-Hit version 4.5.4 [64] and CAP3 [65] for multiple alignments and consensus building. The threshold was set as identity equal to 1 in CD-Hit, the minimal overlap length and identity equal to 100 bp and 99% in CAP3.

### Gene Annotation and Ontology

The selected assembly contigs were used as queries against the NCBI zebrafish protein database, the UniProtKB/SwissProt database and the non-redundant (nr) protein database using the BLASTX program. The cutoff E-value was set at 1e-5 and only the top gene id and name were initially assigned to each contig. Gene ontology (GO) annotation analysis was performed using the zebrafish BLAST results in Blast2GO version 2.6.3 [66]. The zebrafish BLAST result or the nr BLAST result was imported to BLAST2GO. The final annotation file was categorized with respect to Biological Process, Molecular Function, and Cellular Component at level 2.

### Identification of differentially expressed contigs

The high quality reads from each sample were mapped onto the Trinity reference assembly using CLC Genomics Workbench software. During mapping, at least 95% of the bases were required to align to the reference and a maximum of two mismatches were allowed. The total mapped reads number for each transcript was determined and then normalized to detect RPKM (Reads Per Kilobase of exon model per Million mapped reads). The proportions-based test was used to identify the differently expressed genes between fed and fasted group with three replicates in each group with corrected p-value <0.05 [67]. After scaling normalization of the RPKM values, fold changes were calculated. Analysis was performed using the RNA-seq module and the expression analysis module in CLC Genomics Workbench [68]. Transcripts with absolute fold change values of larger than 1.5 were included in analysis as differently expressed genes.

Contigs with previously identified gene matches were carried forward for further analysis. Functional groups and pathways encompassing the differently expressed genes were identified based on GO analysis, pathway analysis based on the Kyoto Encyclopedia of Genes and Genomes (KEGG) database, and manual literature review.

### Enrichment Analysis

Enrichment analysis of significantly expressed GO terms was performed using Ontologizer 2.0 using the Parent-Child-Intersection method with a Benjamini-Hochberg multiple testing correction [69], [70]. GO terms for each gene were obtained by utilizing zebrafish annotations for the unigene set. The threshold was set as FDR value <0.1.

### Experimental validation—QPCR

Ten significantly expressed genes with different expression patterns were selected for validation using real time QPCR with gene specific primers designed using Primer3 software. Primers were designed based on contig sequences (Table S1). Total RNA was extracted using the RNeasy Plus kit (Qiagen) following manufacturer's instructions. All the cDNA products were diluted to 250 ng/µl and utilized for the quantitative real-time PCR reaction using the SsoFast™ EvaGreen® Supermix on a CFX96 real-time PCR Detection System (Bio-Rad Laboratories, Hercules, CA). The thermal cycling profile consisted of an initial denaturation at 95°C (for 30 s), followed by 40 cycles of denaturation at 94°C (5 s), an appropriate annealing/extension temperature (58°C, 5 s). Results were expressed relative to the expression levels of 18S rRNA in each sample using the Relative Expression Software Tool (REST) version 2009 [71] as described in (14).

## Supporting Information

Figure S1 Gene ontology (GO) term categorization and distribution of differently expressed genes in channel catfish. GO-terms were processed by Blast2GO and categorized at level 2 under three main categories (cellular component, molecular function and biological process).
(TIF)

Table S1 Primers used for QPCR validation (5′ to 3′).
(DOCX)

Table S2 Differentially expressed genes in the gill and skin between fasted and fed channel catfish. Positive/negative values indicate upregulation and downregulation, respectively, in fasted fish relative to the fed control. Included genes showed fold changes of 1.5-fold or greater and corrected p-value <0.05. Annotation is based on the NCBI zebrafish and nr databases.
(XLSX)

Table S3 Summary of GO term enrichment result of GO-based functional categories of significantly expressed genes in channel catfish after fasting. The 1,545 differentially expressed genes were analyzed as the study set in comparison to all the catfish unigenes. P-value ≤0.1 was considered significant. Population count is the number of genes associated with the term in the population set. Study count is the number of genes associated with the term in the study set. GO names were retained only from GO terms of levels >2.
(DOCX)
