# Supplementary figures and images for: Short-Term Feed Deprivation Alters Immune Status of Surface Mucosa in Channel Catfish (Ictalurus punctatus)
Source: PLoS One. 2013 Sep 4;8(9):e74581. doi: 10.1371/journal.pone.0074581 (PMC3762756; doi:10.1371/journal.pone.0074581)

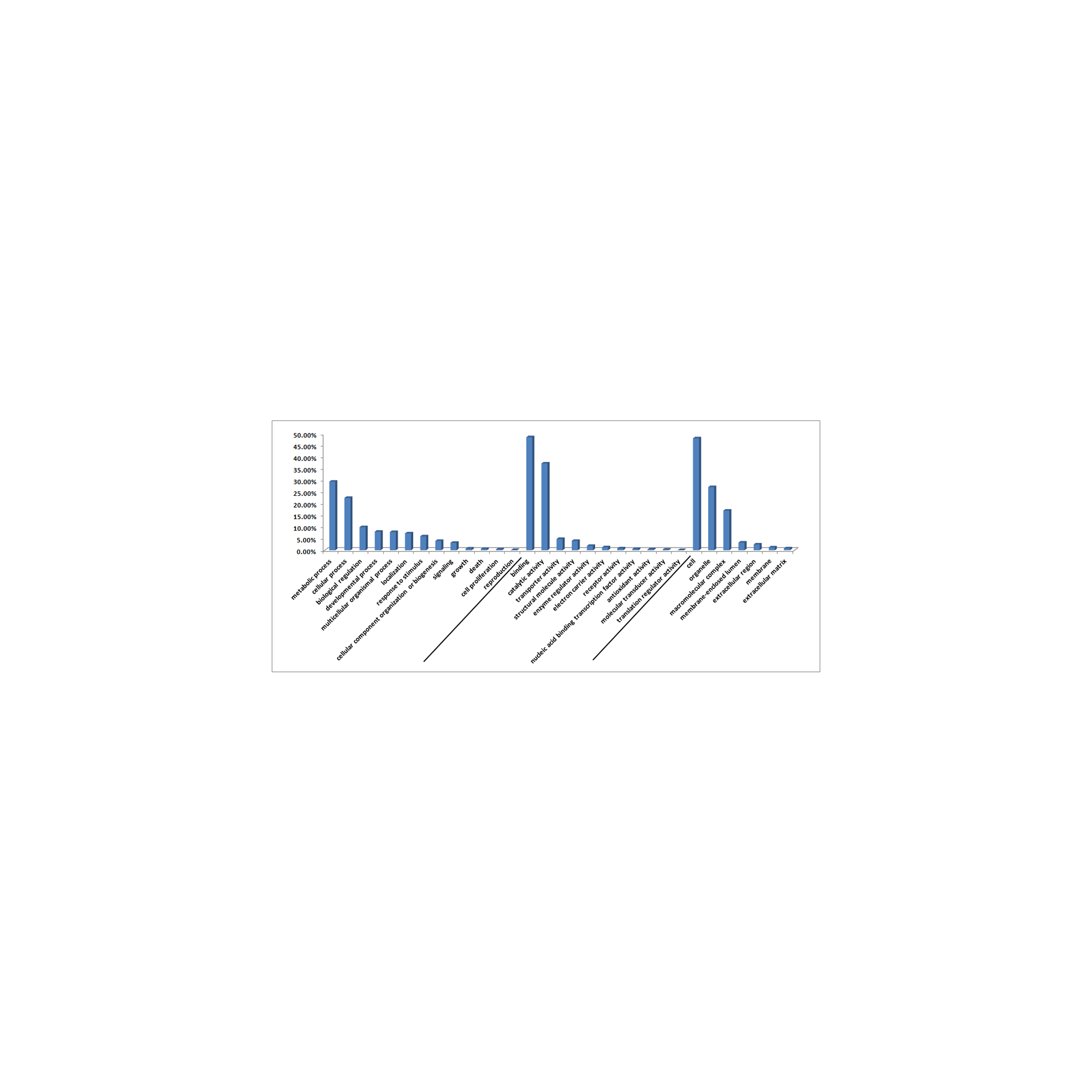

Supplement: Figure S1 — Gene ontology (GO) term categorization and distribution of differently expressed genes in channel catfish. GO-terms were processed by Blast2GO and categorized at level 2 under three main categories (cellular component, molecular function and biological process). (TIF) [file pone.0074581.s001.tif]
