# Supplementary material for: Short-Term Feed Deprivation Alters Immune Status of Surface Mucosa in Channel Catfish (Ictalurus punctatus)
Source: PLoS One. 2013 Sep 4;8(9):e74581. doi: 10.1371/journal.pone.0074581 (PMC3762756; doi:10.1371/journal.pone.0074581)
Supplement: Table S1 — Primers used for QPCR validation (5′ to 3′). (DOCX) [file pone.0074581.s002.docx]

**Table S1**

Primers used for QPCR validation (5’ to 3’)

| Gene | Forward | Reverse |
| --- | --- | --- |
| Anterior Gradient Protein 2 | TCCTTCTTGACCGCAGTCTTGT | AACCTGAGAGCAGCCTGTGAA |
| Complement C4-B | ACTCTGCTGCTTGTAGTTGAAC | GTCGCTCCTGACTCTATAACCA |
| Endothelial Lipase Precursor | CTCACAACGACACCAACGACAT | CCAGCTCACACGGATCTTCAAC |
| Lysozyme G-Like 1 | GCTTCGGACTCATGCAGGTT | TTCTTACTCCAGCGTTGTAGGC |
| Nitric Oxide Synthase 2b, | TCAGCAGATGTCCGATGTCA | AGGAGTTCATTGGTGGAAGGT |
| Phosphomannomutase 2 | GAGATACGCCTGGATGCTCTGT | CGTCGGAGGTTCTGATCTGGAT |
| Stress-Associated Endoplasmic Reticulum Protein 2 | TGATGATGATGGTCCGGTTGTC | ACGAGAAGGCAGCAGTAGGA |
| Taxilin Beta B | ACGGCATCGCTGAAGTGTCT | ACCGCTCAGATCAGAAGCAGTT |
| Twinfilin-2 | ACAGACACTCCACGCTATCACT | GCATCCTCTCCTTAACGCTACA |
| Vitelline Membrane Outer Layer Protein 1 Homolog | AGCCCACAAATCCCAGTTCCA | GATGACACCGCTGCTAACAACA |
| 18S rRNA | GAGAAACGGCTACCACATCC | GATACGCTCATTCCGATTACAG |
