# Supplementary material for: Short-Term Feed Deprivation Alters Immune Status of Surface Mucosa in Channel Catfish (Ictalurus punctatus)
Source: PLoS One. 2013 Sep 4;8(9):e74581. doi: 10.1371/journal.pone.0074581 (PMC3762756; doi:10.1371/journal.pone.0074581)
Supplement: Table S3 — Summary of GO term enrichment result of GO-based functional categories of significantly expressed genes in channel catfish after fasting. The 1,545 differentially expressed genes were analyzed as the study set in comparison to all the catfish unigenes. P-value ≤0.1 was considered significant. Population count is the number of genes associated with the term in the population set. Study count is the number of genes associated with the term in the study set. GO names were retained only from GO terms of levels >2. (DOCX) [file pone.0074581.s004.docx]

**Table S3**

Summary of GO term enrichment result of significantly expressed genes in catfish after fasted. The 1,545 differentially expressed genes were analyzed as the study set in comparison to all the catfish unigenes. P-value ≤ 0.1 was considered significant. Population count is the number of genes associated with the term in the population set. Study count is the number of genes associated with the term in the study set. GO names were retained only from GO terms of levels >2.

| GO ID | GO Name | p-Value(FDR) | Population count | Study count |
| --- | --- | --- | --- | --- |
| GO:0006950 | Response To Stress | 6.46E-11 | 557 | 61 |
| GO:0006520 | Cellular Amino Acid Metabolic Process | 4.53E-04 | 189 | 33 |
| GO:0005198 | Structural Molecule Activity | 2.78E-06 | 347 | 50 |
| GO:0010941 | Regulation Of Cell Death | 0.002554 | 172 | 18 |
| GO:0035556 | Intracellular Signal Transduction | 0.010998 | 749 | 29 |
| GO:0051726 | Regulation Of Cell Cycle | 0.018269 | 149 | 15 |
| GO:0016265 | Death | 0.016633 | 247 | 26 |
| GO:0008219 | Cell Death | 0.022513 | 247 | 26 |
| GO:0006259 | DNA Metabolic Process | 0.038383 | 340 | 39 |
| GO:0022402 | Cell Cycle Process | 0.047922 | 196 | 21 |
